# Nearby arrests and violent crime as predictors of student absenteeism

**DOI:** 10.1371/journal.pone.0323565

**Published:** 2025-07-09

**Authors:** Karl Vachuska

**Affiliations:** 1 Department of Sociology, University of Wisconsin-Madison, Madison, Wisconsin, United States of America; The University of Queensland, AUSTRALIA

## Abstract

This study tests the role of violence and policing in predicting student absences at the school level in New York City. It uses a large dataset on daily attendance over six school years (2013–2014–2018–2019) across all New York City public schools, and operationalizes policing by arrests, and violence by reported violent crime. While much literature focuses on the impact of violence on student outcomes, this study finds that arrests are, in fact, a relatively strong predictor of school absences. Perhaps more importantly, nearby arrests have a uniquely strong association in schools with greater numbers of Black students. In high schools, nearby arrests negatively affect school absences, but only in schools with a high proportion of Black or low-income students. Nearby violence has a small association with absences in K-8 schools, but no significant association with absences in high schools. These findings underscore the need to understand policing’s disparate impact on educational outcomes.

## Introduction

One of the most significant barriers young people have to navigate in space and time in the United States is extensive exposure to policing. Urban and, in particular, Black youth are stopped by police at exceedingly high rates, with this phenomenon beginning at an early age [1]. Black male youth are disproportionately more likely to be stopped by the police compared to their White counterparts. Moreover, they are exceptionally more likely to report aggressive contact with the police. Black men have a 1 in 1000 lifetime chance of being killed by the police, which is 2.5 times the risk of White men [2]. Accordingly, fewer than half of Black youth trust the police, while most White youth do [3]. While these disparities are well-documented at the individual level, this paper focuses on school-level ecological exposure to policing—measured via arrests near schools—to examine whether racial inequalities in absenteeism may also emerge through indirect or ambient forms of police presence.

While policing is a major axis of racial inequality in youth outcomes, another pressing and related challenge is the growing inequality in education [4]. In the US, life outcomes increasingly depend on educational attainment [4,5]. Paramount to understanding educational attainment is understanding primary and secondary school outcomes [6]. Absenteeism is strongly associated with students’ academic performance, educational engagement, and emotional well-being [7,8]. School attendance is closely tied to students’ overall engagement in education [9]. Moreover, regular attendance fosters a sense of belonging, creates opportunities for peer–teacher interactions, and encourages participation in classroom discussions and activities. Understanding what inhibits school attendance is central to unpacking educational inequalities [10].

Chronic absenteeism has a significant impact on children’s learning continuity and their ability to fully engage in classroom activities [10]. This lack of consistent attendance brings far-reaching consequences, particularly for students in their early years. Research indicates that chronic absenteeism not only causes immediate academic setbacks but also has long-term effects on students’ educational trajectories [11,12]. When students frequently miss school, they miss out on crucial instructional time and broader opportunities to develop foundational skills, which may have long-term effects [13]. Consequently, their achievement outcomes in such fundamental domains are compromised. Chronic absenteeism also diminishes educational engagement, limits students’ exposure to learning environments, and hinders their ability to benefit fully from the educational opportunities schools provide. Ultimately, high rates of absence are highly predictive of not being able to graduate from high school [6,7,14].

In addition to the academic consequences, chronic absenteeism also affects students’ socio-emotional well-being [8]. Regular school attendance is fundamental for the formation of children’s social skills and emotion regulation and can also help foster a sense of connectedness within the school community. Ultimately, schools serve as an important social resource for students, especially the most disadvantaged, for whom schools can provide otherwise unavailable social services [15]. Notably, attendance rates differ widely based on race and socioeconomic status [16]. Past research has found that students of color and low-income students tend to experience disproportionately higher rates of absenteeism compared to their peers [17].

This study investigates the association between both policing and violence with school absenteeism, operationalized here as a student missing a school day with or without an excuse. This study aims to explore how nearby arrests and violent crimes affect students’ absence from school, with a specific focus on *school-adjacent* policing and violence, not residential neighborhood exposure. It also investigates whether the school’s racial and socioeconomic composition moderates this relationship. Furthermore, it analyzes this relationship in high schools and K-8 schools to test whether the associations between nearby arrests and nearby violence with school absenteeism is heterogeneous based on the school level. I hypothesize that high school students are more likely to be absent from school in the wake of intense policing, as older students are more likely to have direct contact with the criminal justice system. However, if the effect of intense policing on absenteeism is indirect and operates through stress and familial challenges, one may expect younger students to be strongly affected by nearby arrests.

By leveraging big data over many schools and many school days, I aim to shed light on the precise short-term ecological correlates between violence, policing, and student absenteeism. This study addresses three research questions. First, it examines whether nearby arrests and nearby violent crimes can broadly predict absenteeism in New York City (NYC) public schools. As the NYC public school system is the largest school district in the United States [18], as well as one of the most diverse [19], it is an ideal site to study student absenteeism. Second, this study examines high schools and K-8 schools separately as they may relate differently to policing and violence. Third, it investigates whether the marginal effect of nearby arrests and nearby violence on absenteeism is heterogeneous based on the school’s racial composition and students’ socioeconomic status. Ultimately, this analysis aims to contribute to an improved understanding of how neighborhood violence and neighborhood policing, two often-conflated concepts, are independently associated with student absenteeism. I would emphasize the goal of the analysis is not to compare the size of the association of violence with absences with the size of the association of arrests with absences, but rather to distinguish their independent contributions to absenteeism and to understand how each varies across schools with different racial and socioeconomic compositions.

Regarding school-level drivers of academic disparities, research has extensively focused on the impact of violence, particularly its effects on attendance and academic achievement. Exposure to neighborhood or school-adjacent violence has been shown to harm students’ academic outcomes by undermining perceptions of safety, increasing stress, and disrupting routines [9–11,17]. These effects often accumulate over time, especially for students in persistently violent environments, leading to growing achievement gaps [11]. A key mechanism linking exposure to violence and academic outcomes is absenteeism. For example, Burdick-Will et al. find that students exposed to violent crime during their commute are significantly more likely to be absent, especially when relying on public transportation [13]. Similarly, Balfanz and Byrnes argue that school safety concerns are strongly associated with chronic absenteeism. Together, these findings suggest that violence in students’ environments—whether experienced directly or indirectly—can play a substantial role in shaping attendance patterns and educational inequality [10].

While spatial inequality in exposure to violence has been implicated in educational inequality, public safety interventions—often in the form of increased policing—have been proposed as a means to reduce violence and improve school quality [20]. However, most existing research focuses either on residential exposure to policing or the role of police officers within schools themselves. This study instead examines arrests that occur around schools, which may shape the school climate and student experience even if students do not live nearby or have direct contact with police. Importantly, research suggests that over-policing disproportionately affects Black youth and may have cumulative consequences for educational outcomes [21–23]. Extended exposure to neighborhood policing has been linked to lower high school graduation rates, especially among students of color, raising important questions about how arrests in the vicinity of schools may influence absenteeism and inequality.

While prior work has documented the disproportionate exposure of Black communities to aggressive and racialized policing, this study focuses specifically on ecological exposure—the frequency of arrests and violent incidents in the area surrounding a school—and its relationship to school-level absenteeism. Rather than measuring individual-level experiences of police contact or in-school policing, this paper investigates how the surrounding environment of a school, as shaped by policing and violence, may influence student attendance patterns. This ecological perspective provides a novel lens to understand the broader institutional and community factors that affect educational outcomes.

Previous research has observed correlations in terms of the relationship between contact with police and absenteeism at an individual level [24]. Students who are arrested suffer worse attendance because of their arrest. Recent research suggests that institutional mechanisms drive the relationship between arrests and attendance. Mark and Geller found that arrests directly contribute to absences because of school suspensions and court appearances, not because of the students deciding to skip school [25]. Del Toro et al. and Del Toro and Wang found that police contact reduces academic adjustment and school engagement while increasing defiant behaviors. Furthermore, institutional trust and psychological distress mediate the association between police contact and student outcomes [21,26–28]. Other research posits that policing impacts interaction with institutions (attendance) through avoidance [29]. Additionally, scholars have observed that arrests tend to have indirect effects on attendance for younger students. Some scholarship suggests that the arrest of a parent can result in a short-term increase in absenteeism [30]. These effects are likely to be greater in younger students who are less independent.

While some research has looked at the individual-level effect of arrests on absenteeism [25], and other research has looked at the effect of large-scale policing interventions [22], little research has explored the ecological school-level impact of temporal variation in both violence and arrests on school attendance. Notably, several structural mechanisms suggest that the ecological impact of arrests may be heterogeneous in terms of school racial composition. As a result of longstanding structural racism, as well as police violence tending to be concentrated in low-income, Black neighborhoods [31], Black communities have more collective trauma and may respond differently to police contact compared to other communities [32]. In addition, the nature of policing tends to vary between Black and White communities, with Black communities much more likely to be subject to violent, more intensive police activity when policing does occur [33]. Consequently, the impact of policing on student absenteeism may vary ecologically in terms of racial composition rather than simply individually in terms of race.

Ultimately, the relationship between violence, arrests, and level of attendance is further complicated by the fact that acts of violence often directly result in arrests. Periods of localized violence are inevitably strongly associated with a number of arrests. However, the criminalization of young men of color often also means that policing and arrests rarely occur as a direct result of an act of violence but instead are in response to non-violent criminal activity or serving warrants [34,35]. In New York City, the vast majority of arrests are for non-violent offenses. Broadly, however, the same neighborhoods that experience high levels of violence are likely to experience high levels of policing. However, the timing of the incidence of violence versus the incidence of overreaching policing practices may be somewhat distinct. Disentangling the ecological effects of arrests from those of violence is therefore critical both analytically—since they may reflect different underlying processes—and for policy, as strategies aimed at reducing violence through increased policing may inadvertently harm educational outcomes. By drawing a more precise ecological distinction between violence and policing, scholars and policymakers can move toward better isolating the impact of policing on communities.

This study builds on prior work showing that indirect exposure to violence can harm students even when they are not directly victimized. I extend this logic to policing, proposing that arrests near schools may affect students by disrupting daily routines, eroding institutional trust, or generating fear and stress, particularly in historically over-policed communities. Rather than focusing on direct victimization or familial arrest, this paper emphasizes the ecological exposure to violence and policing in school surroundings as a potential mechanism contributing to disparities in student attendance.

### Data

All data involved in this study was downloaded from the NYC Open Data Portal. Attendance data of public schools in NYC from the 2013–2014 to the 2018–2019 school year were obtained from the New York City Open Data Repository. These datasets contain daily attendance records of all public schools in NYC. Each dataset contains observations of school-day combinations specifying the number of students who were enrolled in a school on a given day and the number of students who were absent on that given day. My primary outcome variable of interest is the number of absences reported in each NYC public school on each school day, from the 2013–2014 to the 2018–2019 school year. Absences reflect lost instructional time that may negatively impact broader educational outcomes. Schools are the unit of analysis in this panel study to explore variation in absences between schools over time. The total sample of schools over this period is 9725 (~1600 schools per year over 6 years), which is reduced to 9194 after excluding schools that cannot be matched to a specific location. Daily attendance datasets are maintained by the New York City Department of Education (DOE) to provide daily information on student attendance in DOE schools. The dataset contains information on the number of students enrolled, present, and absent on each day of school, categorized by the School Identification Number (DBN) and the calendar date. In the dataset, DBN is a unique identification number for each school by which other datasets are linked. Daily absences, as defined here, refer to both excused and unexcused absences that result in a student not being present in school for the required 3.5 hours needed to be considered present on a given school day. Suspended students who remain enrolled at their school are not considered absent as long as they report being present to receive alternative instruction. The geographical coordinates of the schools were sourced from the School Locations dataset. About 5% of schools in the Attendance dataset had no match in the School Locations dataset and, therefore, were excluded from the analysis.

In addition to the aforementioned independent variables, I examine how school attributes modify the associations between nearby arrests and nearby violence with student absenteeism. In particular, I consider the potential role of school racial and socioeconomic composition. Data on school racial and socioeconomic composition is obtained from yearly releases of the Demographic Snapshot datasets released by the NYC DOE. This data is at the year level, with one observation for each school per school year. School racial composition is measured based on the number and percentage of students at a school that identify as non-Hispanic Black, non-Hispanic White, Hispanic (of any race), non-Hispanic Asian, or another or more than one group. Given the particular role that violence and policing may play in the lives of Black students, I particularly focus on racial heterogeneity in terms of percentage Black. I additionally explore heterogeneity in terms of socioeconomic status. I measure school socioeconomic status as the percentage of students in poverty, which the NYC DOE defines as being eligible for reduced or free lunch, or as being eligible for Human Resources Administration benefits. I test coefficient modification in terms of school racial and socioeconomic composition using interaction terms between the independent variables and measures of school composition, and I assess the significance of the coefficient using a p-value threshold of 0.05. Lastly, I explore grade-level heterogeneity by employing stratified models looking at two groups of schools. I define high schools as schools that serve any grade in grades 9−12, and define K-8 schools as all schools that do not.

Data on nearby arrests that occurred within a 1000-meter radius of each school were collected from the NYPD Historical Arrests Data dataset. I employ arrests near schools as a proxy for policing, which I hypothesize to be associated with increased absences. I operationalize nearby arrests as the number of arrests within 1000 meters of each school on the day preceding a given school day. I choose a one-day time lag in alignment with past research on the effect of police contact on academic disengagement [21]. The NYPD Historical Arrests Data dataset contains a comprehensive record of every arrest made by the NYPD between 2006 and 2022. This dataset is produced from manual extracts of NYPD data and is reviewed quarterly by the Office of Management Analysis and Planning before publication. The data are provided by the NYPD and owned by NYC OpenData, a repository of public data managed by the City of New York. Each observation in the dataset shows an arrest made by the NYPD in NYC and includes information about the location and date of arrest.

The second of two independent variables of interest is the level of violence in a particular area surrounding a school. I operationalize nearby violence as the number of reported violent crimes within 1000 meters of each school on the day preceding a given school day. I obtain data on reported violent crimes from the NYPD Complaints Incident Level Data dataset, which provides information such as the date and time of the crime incidents reported in NYC to NYPD from 2001 to the present day. This dataset is also produced from manual extracts of NYPD data and is reviewed quarterly by the Office of Management Analysis and Planning before publication. The data are provided by the NYPD and owned by NYC OpenData. A complaint involving multiple offenses is classified based on the most serious offense. The Report Date indicates the date on which the incident was reported to the NYPD. The data show criminal offenses according to the definitions stated in the New York State Penal Law, which are more distinctly defined than those in the FBI Uniform Crime Report. The following types of violent crimes in NYC are included in the analysis: Assault 3 and Related Offenses, Felony Assault, Robbery, and Homicide. It is important to recognize that this measure of violence accounts only for violent crimes that have been reported to the police and likely misses a substantial number of acts of violence which occurred but were not reported to the police [36–39].

The choice to use a spatial bandwidth as opposed to another form of geographical boundaries, such as a school catchment zone, was motivated by multiple factors. First of all, school catchment areas are inconsistently used in New York City, and essentially non-existent at the high school level in New York City, making their application both challenging and problematic. Additionally, school-level data on how far students travel to attend school makes other spatial measures that capture residential exposures infeasible. Moreover, the choice to focus on arrests and violence proximal to schools as opposed to residential exposure is in line with past research [13], which suggests school attendance is more closely related to school-related exposures. In terms of using a bandwidth surrounding schools, past research in this area has leveraged a variety of distances [40]. Since I do not believe any single bandwidth is perfectly motivated, the goal of the analysis is to test the robustness of the findings to different bandwidths. With that in mind, analyses of the data and specific school cases do support the idea that a slightly larger bandwidth is preferable for a couple of reasons. For one, some schools in NYC have large enough school grounds that a few hundred meters does not even reach across the school grounds. The location of schools is a single geographical point—if the bandwidth cannot reach from that point to another location on the school grounds, arrests or violence that occur at some schools could be missed, which is especially problematic. Additionally, past research suggests that criminal activity associated with schools does spill over to residential areas adjacent to schools [41,42]. Additionally, another advantage of a slightly larger bandwidth is that the signal of arrests or violence becomes stronger. With very small bandwidths, the average number of arrests or violent crimes for a given school could average less than one per day. While seeing how a single violent crime or arrest that is very proximal to school is associated with attendance, such an analysis omits violent crimes or arrests that are right outside the bandwidth (potentially just a block away). Ultimately, I employ robustness checks of 500-meter and 250-meter bandwidths in addition to using a bandwidth of 1000 meters for the primary analysis.

### Statistical analysis

Table 1 presents the summary statistics. A series of Poisson regression models were used to examine the individual and combined associations of nearby arrests and violent crimes with school absenteeism in NYC public schools. Poisson regression assumes the outcome variable follows a Poisson process and is a good choice for modeling the number of students who show up to school on a given day because the outcome variable is a non-negative count. Poisson regression assumes the outcome variable is logarithmically associated with the predictor variables and is estimated using maximum likelihood. This methodological choice is in line with past research [43]. The primary model was as follows:

**Table 1:** Summary statistics.

Variable	Mean	SD	Min	Pctile[25]	Pctile[75]	Max
Enrolled	600.2	486.619	1	321	707	5955
Absent	51.1	61.436	0	22	60	3332
Arrests	7.1	8.012	0	1	10	246
Violence	1.9	2.19	0	0	3	28
High School	0.7	0.451	0	0	1	1
Perc. Black	25.4	26.855	0	1.9	38.8	98.7
Perc. Poverty	62.7	33.246	0.038	45.3	88.1	100


$$\ln(\mu_{ij})=\beta_{1}*V_{ij}+\beta_{2}*P_{ij}+{∆}_{i}+\nabla_{j}+\ln(ENR_{ij})$$

where *i* denotes a specific school on a particular day (*j*). The outcome variable (*μij*) is the expected number of students absent from the school. β1 and β2 are the coefficients of the number of nearby violent crimes (*Vij*) and the number of nearby arrests (*Pij*), respectively. These coefficients measure the association between nearby violent crimes and arrests and student absenteeism. *∆i* denotes school-level fixed effects to account for unobserved characteristics that vary across schools but remain constant over time. ∇*j* captures day-level fixed effects to account for unobserved factors that vary across days but remain constant across schools. Finally, *ln(ENRij)* denotes an offset term—the natural logarithm of the number of students enrolled in the school (*ENRij*). This variable serves as a control for the size of the student population, adjusting for enrollment in predicting student absences. The coefficients (*β1* and *β2*) quantify the association between nearby violent crimes and arrests and student absences while controlling for school- and day-level fixed effects. Day-level fixed effects account for unique combinations of day, month, and year. Given the logarithmic scale of the Poisson model, unit increases can be interpreted by exponentiating them and comparing them multiplicatively to the value 1. For example, a coefficient of 0.025 for a given variable would indicate that a one-unit increase in that variable is associated with *exp(0.025)* or *~1.0253* times more absences (a 2.53% increase). It is important to note that because violence is measured as violent crimes reported to the police, the coefficient estimate may be misestimated to the extent to which actual violence is underreported. To address the possibility of uniform underreporting, I interpret coefficient estimates in terms of standard deviation changes. I include a more thorough discussion of potential underreporting in the discussion section.

I include all public schools in NYC whose attendance data can be linked with location-related data from the 2013–2014 to the 2018–2019 school year. All school days between September and June are included in the analysis. The dependent variable is the number of students absent from school on a given date. The independent variables are the number of violent crimes and the number of arrests within a 1000-meter radius of the school on the day prior. Nearby violent crimes are operationalized as reported incidents described as “Felony assault,” “Assault 3 and related offenses,” “Robbery,” and “Murder & Non-Negl. Manslaughter”. The distance from school to the crime/arrest is calculated using the geodist package in R. Arrests include all arrests, regardless of their type. I do employ a robustness check on the main results where I count only arrests from non-violent offenses. Other school-level variables include the percentage of non-Hispanic Black students and the percentage of low-income students. These variables are taken directly from the Demographic Snapshot of NYC public schools.

**Fig 1:**
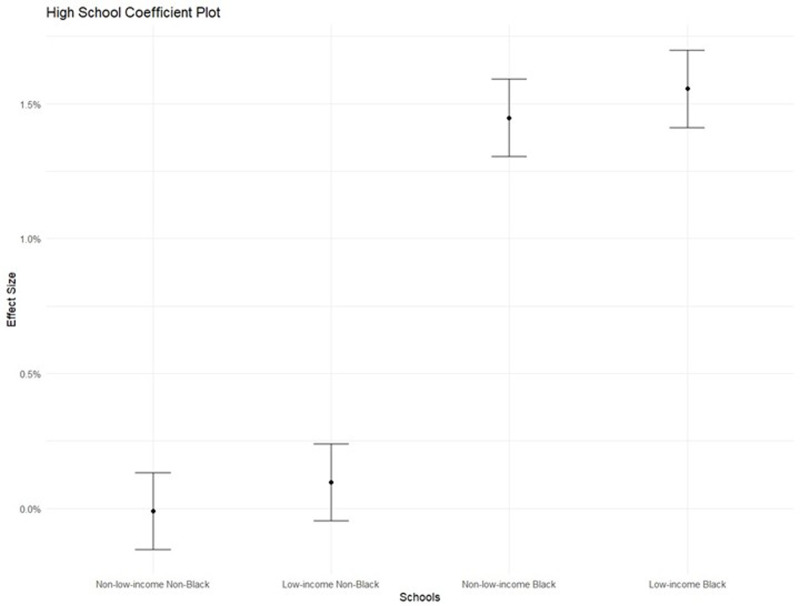
Coefficient plot: High school.

**Fig 2:**
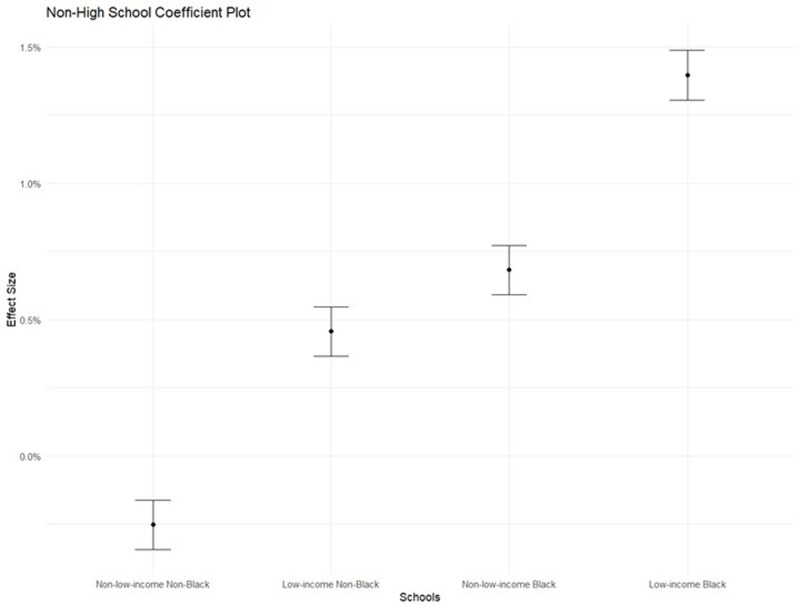
Coefficient plot: K-8 school.

The first two models I present contain one predictor each (in addition to school and day-fixed effects and an enrolled offset term, which are included in all models) in estimating the count of student absences. The first and second models contain the number of nearby arrests and nearby violent crimes, respectively. The third incorporates both predictors. The fourth model tests for heterogeneity in terms of school racial composition by incorporating two additional interaction terms between the percentage of students in the school who identify as non-Hispanic Black and nearby arrests and nearby violent crime. The fifth model tests for heterogeneity in terms of school socioeconomic composition by incorporating two additional interaction terms between the percentage of students in the school in poverty and nearby arrests and nearby violent crime. Finally, the sixth model incorporates a combination of interaction terms from the fourth and fifth models based on which terms are statistically significant in each of the two models. This selection was performed in order to construct a parsimonious and interpretable final model, given the potential for multicollinearity between racial and socioeconomic composition. Given the correlation between socioeconomic and racial composition, this sixth model allows me to explicitly test whether school racial or socioeconomic composition drives heterogeneity. To facilitate the interpretation of these interactions, I generate predicted absenteeism levels under different scenarios using model estimates. Specifically, I calculate the marginal effect of a 10-arrest increase for schools with varying racial and socioeconomic compositions (e.g., 10% vs. 80% Black students and 10% vs. 50% poverty rate). The resulting plots in Figs 1 and 2 visually depict how the effect of arrests depends on school context. This visualization strategy is especially helpful given the complexity of interpreting interaction terms in non-linear models like Poisson regressions.

To explore heterogeneity in terms of school level, I run the aforementioned sequence of models on high schools and K-8 schools. Stratified summary statistics for high schools, K-8 schools, predominantly Black schools, predominantly low-income school, predominantly Black and low-income schools, predominantly non-Black schools, predominantly non-low-income school, and predominantly non-Black and non-low-income schools can be found in the appendix (S1 to S8 Tables in S1 File).

The significance of all coefficients is assessed using a p-value threshold of 0.05. The central assumption behind all models is that no unobserved confounding exists. While eliminating the presence of unobserved confounding factors is impractical, two-way fixed effects models are one of the most commonly employed tools for deriving estimates of causal effects from observational data [44]. While I do not make causal claims, the predictive power of these models can still be a highly important tool for policy. The predictive results of these models may assist policymakers and school administrators to effectively allocate resources to mitigate absenteeism at specific schools and at specific points in time. Predictive findings additionally open the door for subsequent research to further explore the specific pathways through which violence and policing are related to school attendance.

### Sensitivity analysis

To evaluate the robustness of the main findings, I conduct a series of sensitivity analyses. First, I test whether results hold when disaggregating school racial composition into multiple racial groups, rather than focusing solely on percentage Black. Second, I re-estimate the main models including quadratic terms for percentage Black and percentage in poverty to assess potential non-linearities in their relationship with absenteeism. Third, I re-estimate models measuring arrests using only non-violent arrests to ensure the results are not simply capturing the effect of violent crime arrests. Fourth, I test whether including local NYPD 311 complaint data alters the estimated associations, helping to account for broader signals of demand for police. Fifth, I replicate the main models using ordinary least squares (OLS) instead of Poisson regression to test sensitivity to model specification. Sixth, I standardize arrest and violence exposure measures within schools to isolate across-time within-school variation. Finally, I assess the robustness of the findings to alternative spatial definitions by re-running the main models using 500-meter and 250-meter radii instead of the primary 1000-meter radius.

## Results

### All schools

Table 2 presents the results of examining nearby violent crimes and nearby arrests as predictors of school absenteeism in all NYC public schools. Model 1 comprised a Poisson model that included two-way school- and day-level fixed effects, with the number of arrests within a 1000-meter radius of the school the previous day as the independent variable. The significance level of the coefficient revealed that nearby arrests made the previous day are significantly associated with the number of students absent from school the next day. More specifically, a one-standard-deviation increase in the number of nearby arrests the previous day increased the number of school absences by 0.7% (coef: 0.00104; SE: 0.00010). This result alone does not imply a specific mechanism behind this association. Arrests might be a proxy for criminal activity or intense policing. Moreover, arrests might increase school absences owing to students getting arrested, facing intense policing, attempting to evade surveillance, or experiencing communal or family disorder.

**Table 2:** Poisson models predicting school absenteeism in all schools.

	Model 1	Model 2	Model 3	Model 4	Model 5	Model 6
Nearby Arrests	0.00104***		0.00096***	−0.00001	0.00012	−0.00004
	(0.00010)		(0.00010)	(0.00014)	(0.00021)	(0.00021)
Nearby Violence		0.00177***	0.00135***	0.00153***	0.00060	0.00133***
		(0.00025)	(0.00025)	(0.00036)	(0.00051)	(0.00025)
Nearby Arrests X Perc. Black				0.00003***		0.00003***
				(0.00000)		(0.00000)
Nearby Violence X Perc. Black				−0.00001		
				(0.00001)		
Nearby Arrests X Perc. Poverty					0.00001***	0.00000
					(0.00000)	(0.00000)
Nearby Violence X Perc. Poverty					0.00001	
					(0.00001)	
N	1587887	1587887	1587887	1587887	1587887	1587887
AIC	16589216.80170	16590088.98576	16588874.58888	16587879.14059	16588638.12213	16587884.78956
BIC	16713849.91442	16714722.09849	16713519.97952	16712549.08705	16713308.06860	16712554.73603
Pseudo R2	0.77118	0.77117	0.77119	0.77120	0.77119	0.77120

*** p < 0.001; ** p < 0.01; * p < 0.05.

All models include school and date fixed effects and an offset term equal to the logged number of students reported to be enrolled in the school on the given day. Standard errors are clustered by school. Coefficients can be interpreted as the effect of a one-unit change in the variable on the logged count of students absent from school on a given day. Perc. Black refers to the percentage of students in the school that identify as non-Hispanic Black. Perc. Poverty refers to the percentage of students in the school that are considered in poverty. Nearby arrests refer to the number of arrests within 1000 meters of the school on the day prior. Nearby violence refers to the number of violent crimes within 1000 meters of the school on the day prior.

Model 2 included a Poisson model with two-way school- and day-level fixed effects. The independent variable was the number of violent crimes reported within a 1000-meter radius of the school the previous day. The correlation between violent crimes reported within a 1000-meter radius and the number of arrests is 0.51. The significance level of the coefficient revealed that nearby violent crimes the previous day are significantly associated with the number of school absences the next day. More specifically, a one-standard-deviation increase in the number of nearby violent crimes recorded the previous day increased the number of school absences by 0.4% (coef: 0.00177; SE: 0.00025).

Model 3 combined the independent variables of Models 1 and 2. The results revealed that both nearby arrests and violent crimes significantly predict school absences. Notably, the marginal effect of nearby arrests and violent crimes was not substantially attenuated relative to the independent models. More specifically, a one-standard-deviation increase in the number of nearby arrests and violent crimes the previous day increased the number of school absences by 0.7% (coef: 0.00096; SE: 0.00010) and 0.4% (coef: 0.00136; SE: 0.00026), respectively. Surprisingly, while nearby violent crimes and arrests are strongly correlated, the results of this model indicated that their association with school absences appears primarily independent.

Model 4 replicated Model 3 but added an interaction term of the percentage of non-Hispanic Black students in the school and the number of nearby arrests and an interaction term of the percentage of non-Hispanic Black students in the school and the number of nearby violent crimes. Notably, the coefficient of nearby arrests substantially decreased and became insignificant in this model. The coefficient of nearby violent crimes remained close to its value in Models 2 and 3 but increased slightly. Interestingly, the coefficient of the interaction term of nearby arrests and the percentage of Black students in the school was positive and highly significant. All else being equal, the coefficient suggested that a one-standard-deviation increase in arrests near a school that has 80% Black students increases the number of absences by 2.4% (coef: 0.00003; SE: 0.00000). This is much greater than at a school with an average share of Black students (25.4%), where a one-standard-deviation increase in arrests is associated with only 0.3% more arrests. The coefficient of the interaction term of nearby violent crimes and the percentage of Black students in the school was negative and insignificant. Overall, this model showed that the association between nearby violent crimes and school absences is somewhat ubiquitous across all schools, regardless of their racial composition. Distinctly, the interaction term of the percentage of non-Hispanic Black students and the number of nearby arrests revealed that the association between nearby arrests and school absences is non-existent in schools with small proportions of Black students but significant in schools with large proportions of Black students.

Model 5 was analogous to Model 4 but included an interaction term of the percentage of low-income students in the school and the number of nearby violent crimes, as well as an interaction term of the percentage of low-income students in the school and the number of nearby arrests. The results of this model were similar to those of Model 4, with the coefficient of nearby arrests being insignificant. Meanwhile, the coefficient of the interaction term of the percentage of low-income students in the school and the number of nearby arrests was positive and highly significant. Notably, the coefficient of nearby violent crimes was attenuated to insignificance in this model. Moreover, the coefficient of the interaction term of the percentage of low-income students in the school and the number of nearby violent crimes was also insignificant, suggesting that this interaction term might not be appropriate. Ultimately, this model suggests that the association between arrests and student absenteeism is conditional on school socioeconomic composition. This aligns with recent research that suggests that socioeconomic disadvantage is uniquely relevant to understanding individuals’ and communities’ relationships with law enforcement.

Model 6 explicitly tested whether the association between nearby arrests and school attendance depends on the percentage of Black or low-income students in the school, two variables that are strongly correlated in NYC public schools. The results strongly suggest that it is racial composition, not socioeconomic status, that matters in the association between nearby arrests and school attendance. The coefficient of both nearby arrests and the interaction term of nearby arrests and the percentage of low-income students in the school was entirely insignificant. Meanwhile, the coefficient of the interaction term of nearby arrests and the percentage of Black students in the school was positive and highly significant.

Supplementary analyses suggest that schools with higher proportions of Hispanic students also have a greater association between arrests and student absences (S9 Table in S1 File). Additionally, the aforementioned analyses are robust to a quadratic specification for percentage Black and percentage in poverty (S10 Table in S1 File). These findings suggest that the association between arrests and absences continuously increases with the share of students who are Black or in poverty, but this slope becomes more gradual as these values approach 100%. The main results are also robust to excluding arrests for violent crimes in the nearby arrests measure (S11 Table in S1 File). Additionally, the main results are robust to controlling for nearby 311 calls routed to NYPD (S12 Table in S1 File), suggesting that adjusting for local demand for policing does not modify the results substantially. In terms of functional form, the main results are also robust to using OLS estimation to predict the logged fraction of students absent (S13 Table in S1 File). The results are additionally robust to measuring nearby arrests and nearby violence using a school-standardized measure that accounts for variation in typical numbers of arrests and violent crimes near schools (S14 Table in S1 File). Finally, the findings are consistent also using a 500-meter (S15 Table in S1 File) and 250-meter (S16 Table in S1 File) bandwidth. While the marginal effect of a one-standard-deviation increase in nearby arrests is highly statistically significant (p < .001) across all three bandwidths, the effect shrinks with smaller bandwidths—a 0.7% increase in absences with 1000-meters, a 0.4% increase with 500-meters, and a 0.1% increase with 250-meters. The marginal effect of a one-standard-deviation increase in nearby violence is highly statistically significant (p < .001) across all three bandwidths and also shrinks with smaller bandwidths, a 0.4% increase with 1000-meter, a 0.2% increase with 500-meters, and a 0.2% increase with 250-meters. These smaller associations are potentially due to the increasingly weaker signal in the measure of arrests and violence as the bandwidth shrinks.

Overall, the main results reported in Table 2 coupled with the robustness checks, provide strong evidence of how violence and policing are associated with student attendance. Broadly, nearby violence was mildly associated with absenteeism in all types of schools, independent of schools’ racial composition and students’ socioeconomic status. Distinctly, nearby arrests were not associated with absenteeism in schools with small percentages of Black students but strongly related to absenteeism in schools with large percentages of Black students.

### High schools vs. K-8 schools

Table 3, Panel A includes the same model sequence as Table 2, but the analytic sample is restricted to high schools only. The results mostly replicated the main results with a few exceptions. Little to no relationship between nearby violent crimes and school absences was observed; only the relationship between nearby arrests and school absences was observed. Similar to the results obtained from the main models, the relationship between nearby arrests and school attendance was significant depending on the percentage of Black students in the school. The size of the combined marginal effect, as revealed by Model 4, suggests that a one-standard-deviation increase in arrests near a high school that has 80% Black students is associated with a 1.4% increase in school absences (Nearby Arrests coef: 0.00008; SE: 0.00025 and Nearby Arrests X Perc. Black coef: 0.00003; SE: 0.00001).

**Table 3:** Panel A. Poisson models predicting school absenteeism in high schools. Panel B. Poisson models predicting school absenteeism in K-8 schools.

	Model 1	Model 2	Model 3	Model 4	Model 5	Model 6
**Panel A. Poisson models predicting school absenteeism in high schools.**						
Nearby Arrests	0.00098***		0.00094***	0.00008	0.00081*	0.00066
	(0.00018)		(0.00018)	(0.00025)	(0.00038)	(0.00037)
Nearby Violence		0.00095*	0.00055	0.00167**	0.00074	0.00053
		(0.00041)	(0.00042)	(0.00061)	(0.00086)	(0.00042)
Nearby Arrests X Perc. Black				0.00003***		0.00003***
				(0.00001)		(0.00001)
Nearby Violence X Perc. Black				−0.00004*		
				(0.00002)		
Nearby Arrests X Perc. Poverty					0.00000	−0.00001
					(0.00000)	(0.00001)
Nearby Violence X Perc. Poverty					0.00000	
					(0.00001)	
N	473651	473651	473651	473651	473651	473651
AIC	4948818.65694	4949180.07854	4948802.82810	4948559.16501	4948805.10693	4948589.48550
BIC	5010413.33486	5010774.75647	5010408.57426	5010187.04762	5010432.98953	5010217.36810
Pseudo R2	0.77719	0.77718	0.77720	0.77721	0.77720	0.77721
**Panel B. Poisson models predicting school absenteeism in K-8 schools.**						
Nearby Arrests	0.00106***		0.00095***	−0.00004	−0.00014	−0.00031
	(0.00012)		(0.00013)	(0.00017)	(0.00026)	(0.00026)
Nearby Violence		0.00208***	0.00166***	0.00151***	0.00051	0.00165***
		(0.00030)	(0.00031)	(0.00044)	(0.00060)	(0.00031)
Nearby Arrests X Perc. Black				0.00004***		0.00003***
				(0.00000)		(0.00001)
Nearby Violence X Perc. Black				0.00001		
				(0.00001)		
Nearby Arrests X Perc. Poverty					0.00002***	0.00000
					(0.00000)	(0.00000)
Nearby Violence X Perc. Poverty					0.00002*	
					(0.00001)	
N	1114236	1114236	1114236	1114236	1114236	1114236
AIC	11564486	11564938	11564132	11563355	11563832	11563340
BIC	11667197	11667649	11666855	11666101	11666578	11666086
Pseudo R2	0.77019	0.77018	0.7702	0.77022	0.77021	0.77022

*** p < 0.001; ** p < 0.01; * p < 0.05.

All models include school and date fixed effects and an offset term equal to the logged number of students reported to be enrolled in the school on the given day. Standard errors are clustered by school. Coefficients can be interpreted as the effect of a one-unit change in the variable on the logged count of students absent from school on a given day. Perc. Black refers to the percentage of students in the school that identify as non-Hispanic Black. Perc. Poverty refers to the percentage of students in the school that are considered in poverty. Nearby arrests refer to the number of arrests within 1000 meters of the school on the day prior. Nearby violence refers to the number of violent crimes within 1000 meters of the school on the day prior.

Table 3, Panel B also includes the same model sequence as Table 2, but the analytic sample is restricted to K-8 schools only. The results closely replicate the main results. The relationship between nearby arrests and school attendance was found to be insignificant, controlling for the interaction of nearby arrests with the percentage of Black students and the percentage of low-income students in the school. Similar to the results obtained from the main models, nearby violence affected school attendance, but the relationship was not conditional on the school’s racial composition and only slightly conditional on students’ socioeconomic status. The size of the combined marginal effect suggested that a one-standard-deviation increase in arrests near a K-8 school that has 80% Black students and 80% low-income students is associated with a 1.1% increase in school absenteeism (Nearby Arrests coef: −0.00004; SE: 0.00017 and Nearby Arrests X Perc. Black coef: 0.00004; SE: 0.00000).

Figs 1 and 2 present the estimated marginal effect of 10 arrests on school absences relative to no arrests. In these hypothetical schools, a “non-Black” school is 10% Black, while a “Black” school is 80% Black. A “non-low-income” school has a 10% poverty rate, and a “low-income” school has a 50% poverty rate. The results depict how, in non-low-income and non-Black schools, there is little to no marginal effect of arrests. Distinctly, in high schools with a high proportion of Black students or in K-8 schools with a high proportion of low-income or Black students, there is a large and highly significant marginal effect of arrests.

## Discussion

The results of this study highlighted the relationship between nearby arrests and violence and school absenteeism. Nearby arrests significantly predicted school absences, indicating the potential adverse impact of policing on educational outcomes. A one-standard deviation increase in nearby arrests was associated with a 0.7% increase in school absences. Similarly, nearby violent crimes significantly predicted school absences. A one-standard-deviation increase in nearby violent crimes increased school absences by 0.4%. Nearby arrests and violence are independently associated with student absenteeism.

Notably, the association between nearby arrests and absences was stronger in schools with a large proportion of Black students and non-existent in other schools. This finding aligns with prior research that suggests that police contact disparately impacts Black students’ absenteeism in addition to other academic outcomes [21–23,26]. While past research has suggested that arrests impact absenteeism through the direct absence of arrested students [25], the specific results here provide suggestive evidence that the association between nearby arrests and school absenteeism operates through various mechanisms beyond the direct absence of arrested students. The small overall proportion of juvenile arrests and the low likelihood of younger students being arrested indicates that other factors, such as fear, anxiety, disrupted family dynamics, and communal disorder, may contribute to the association between nearby arrests and school absenteeism. Although the lack of individual-level data prevents the interpretation of specific conclusions regarding through what mechanisms arrests impact school attendance, such a finding would align with other research suggesting policing has indirect and long-reaching impacts on children [21–23,26–28,45–51].

Distinct from the association between arrests and school absences, the association between nearby violent crimes and school absences remained consistent regardless of school racial and socioeconomic composition. The association between violent crimes and absences was also found to be entirely non-existent in high schools. While this finding is in alignment with past research that suggests that violence does impact absenteeism, the size and limited nature of the effect is somewhat different [9,11–13].

Overall, the findings of this study highlight the large relationship that nearby arrests have with student absenteeism and, notably, that the relationship appears to be particularly central to Black–White inequalities in student absenteeism. The findings emphasize the importance of considering the associations that both nearby violent crimes *and* nearby policing have with school absenteeism. By showing that the association between nearby arrests and student absence is prominent in schools with a large number of Black students, the findings also highlight the significant role of school racial composition on attendance inequalities. Future research should take advantage of individual-level attendance data and multi-level modeling strategies to better disentangle the factors that drive heterogeneity in the effect of arrests on absenteeism.

The results of this analysis suggest that taking measures to reduce violence and enhance safety in neighborhoods surrounding schools may contribute to improving student attendance, but that such measures might not have as sufficiently strong an effect on school attendance equity compared to addressing the adverse impacts of policing. Given that most public safety efforts to reduce violence operate through increased policing, these efforts may be entirely counterproductive to efforts to reduce student absenteeism as well as possibly further exacerbate racial inequality in student absenteeism. These findings call for policymakers to consider the broader implications and social costs of policing strategies in educational outcomes.

### Limitations

Overall, this study provides robust evidence of the association between violence and policing and school absenteeism. The results provide suggestive evidence of the potential adverse impacts of policing, particularly in terms of attendance at low-income, predominantly Black schools. Despite its utility, this study has some limitations. For one, the modifiable areal unit problem makes this type of spatial analysis potentially sensitive to arbitrarily defined boundaries [52]. Relying on a perfectly delineated set of boundaries, like school catchment areas, is infeasible in New York City, where school catchment areas are inconsistently used and essentially non-existent at the high school level. Additionally, school-level data on how far students travel to attend school is not publicly available, making accounting for residential exposures to violence and arrests even more challenging. Moreover, the choice to focus on arrests and violence proximal to schools as opposed to residential exposure is in line with past research [13]. While I have shown that the main pattern of results is robust to multiple spatial bandwidths, these results should still be interpreted with the modifiable areal unit problem in mind.

Additionally, the dataset I used relies on the reported data of violent crimes. Past research has documented a substantial underreporting of violent crimes relative to the true volume that likely occurs in the United States [36]. This substantial underreporting may lead to a potential misestimation of the associations examined. In the case where violence is underreported at random, this may not be very problematic. If violent crimes are systematically underreported, which past research suggests is likely [37–39], this may substantially bias the main coefficient estimates. For example, if violence is specifically underreported near predominantly Black schools or schools with high shares of students in poverty but not near other schools, heterogeneity estimates for the impact of violence on arrests across schools have the potential of being highly biased. A robustness check which standardizes the measure of violence at the school level (S14 Table in S1 File) partially addresses this concern, though readers should still bear this limitation in mind when interpreting the results.

Additionally, nearby arrests may be an inaccurate proxy for the intensity of policing in a certain area. Also, while my models included both school- and day-level fixed effects, unobserved confounding factors remain a potential source of bias. There may be additional factors that influence both policing practices and violent incidents, as well as school absenteeism. For example, there may be an association between delinquent behavior and truancy [53]. If delinquent behavior drives both arrests and absenteeism, estimates of the effect of arrests on absenteeism could be biased in the absence of controls on delinquent behaviors. Finally, this study is also limited by the inherent constraints of drawing causal conclusions from observational research [44]. Experimental designs implementing public safety alternatives that reduce arrests, such as restorative justice programs [54], may yield better estimates of the causal effect of arrests on school attendance as well as better inform future policies to reduce the racially unequal impact of the criminal justice system on youth outcomes. Quasi-experimental designs may also be useful in yielding stronger causal evidence of the impact of policing on student attendance.

### Recommendations for future research

Future studies should address these limitations through additional data sources and experimental and quasi-experimental designs to strengthen the evidence on policing and violence inequalities and their impact on educational outcomes. Furthermore, while this study focused on the impact of the conditions surrounding schools and exposure to policing, a need exists to explore the effects of exposure to violence and policing in other settings, such as residential areas. The data in this study was limited to aggregated school information, which masks residential information for students. Consequently, measuring and disaggregating between school-proximal exposure to violence and arrests as opposed to residential-proximal exposure to violence and arrests is infeasible. To what extent instances of policing or violence near schools are reflective of school versus residential neighborhood effects remain somewhat ambiguous. Future studies that can leverage richer data should investigate these potentially differential roles. As this study is limited in its data scope, future studies should assess the effects of policing on youth development in other contexts and with individual-level data that can further disentangle effects on schools and on students. Additionally, the nature of data in this study (school-level data) makes drawing conclusions about chronic absenteeism impractical without violating the ecological fallacy. Future research that can explore chronic absenteeism at the individual level may yield clearer insights into that especially important form of absenteeism.

Ultimately, the precise mechanisms underlying the association between nearby arrests and absenteeism remain unclear. However, it seems unlikely that the association is entirely due to the absence of arrested students. Juvenile arrests constitute an extremely small proportion of the arrests in NYC, and students younger than high school age are unlikely to be arrested. Since an association between arrests and absences is observed in both high schools and K-8 schools, this suggests that the association between nearby arrests and school absenteeism likely partially operates through other mechanisms. Prior research suggests that avoidance behaviors, broadly, are a common response to police surveillance [29]. Other research suggests that institutional trust mediates the association between police contact and school-based defiant behaviors [27]. It is also possible that fear and anxiety induced by intense policing can induce absenteeism, as past research has found that psychological distress mediates the association between police contact and school disengagement [21]. Finally, it is also possible that arrest-induced disruption in family dynamics and caretaking drives the relationship between arrests and absences [30]. Future research should more carefully evaluate the precise mechanisms that mediate the relationship between policing and student absenteeism. In addition to mediation, future research should additionally build on the work of Del Toro and Wang [28] to better understand the factors, such as cultural socialization, that may positively moderate the association between policing and student absenteeism.

## Supporting information

S1 File Policing education supplementary information. (DOCX)
