# Supplementary material for: Nearby arrests and violent crime as predictors of student absenteeism
Source: PLoS One. 2025 Jul 9;20(7):e0323565. doi: 10.1371/journal.pone.0323565 (PMC12240335; doi:10.1371/journal.pone.0323565)
Supplement: S1 File — (DOCX) [file pone.0323565.s001.docx]

**Supporting Information for**

Nearby Arrests and Violent Crime as Predictors of Student Absenteeism

**Table S1.** High school summary statistics.

| **Variable** | **Mean** | **SD** | **Min** | **Pctile[25]** | **Pctile[75]** | **Max** |
| --- | --- | --- | --- | --- | --- | --- |
| Enrolled | 647 | 728 | 16 | 318 | 596 | 5955 |
| Absent | 76 | 93 | 0 | 33 | 82 | 3332 |
| Arrests | 8.5 | 8.7 | 0 | 2 | 12 | 246 |
| Violence | 2.1 | 2.2 | 0 | 0 | 3 | 24 |
| High School | 1 | 0 | 1 | 1 | 1 | 1 |
| Perc. Black | 30 | 26 | 0 | 7.3 | 41 | 93 |
| Perc. Poverty | 64 | 31 | 0.16 | 58 | 85 | 100 |

*Summary statistics of schools that served grades 9-12*

**Table S2.** K-8 school summary statistics.

| **Variable** | **Mean** | **SD** | **Min** | **Pctile[25]** | **Pctile[75]** | **Max** |
| --- | --- | --- | --- | --- | --- | --- |
| Enrolled | 606 | 353 | 14 | 342 | 782 | 2391 |
| Absent | 40 | 39 | 0 | 19 | 49 | 1278 |
| Arrests | 6.9 | 7.6 | 0 | 1 | 10 | 171 |
| Violence | 1.9 | 2.2 | 0 | 0 | 3 | 28 |
| High School | 0 | 0 | 0 | 0 | 0 | 0 |
| Perc. Black | 23 | 27 | 0 | 1.3 | 36 | 99 |
| Perc. Poverty | 62 | 34 | 0.038 | 40 | 90 | 100 |

*Summary statistics of schools that did not serve grades 9-12*

**Table S3.** Predominantly Black school summary statistics.

| **Variable** | **Mean** | **SD** | **Min** | **Pctile[25]** | **Pctile[75]** | **Max** |
| --- | --- | --- | --- | --- | --- | --- |
| Enrolled | 435 | 247 | 16 | 254 | 557 | 2146 |
| Absent | 44 | 41 | 0 | 20 | 54 | 1464 |
| Arrests | 7.6 | 7.3 | 0 | 2 | 11 | 171 |
| Violence | 2.1 | 2.1 | 0 | 0 | 3 | 21 |
| High School | 0.3 | 0.46 | 0 | 0 | 1 | 1 |
| Perc. Black | 73 | 12 | 50 | 62 | 83 | 99 |
| Perc. Poverty | 83 | 10 | 42 | 76 | 91 | 100 |

*Summary statistics of schools where more than 50% of students were non-Hispanic Black*

**Table S4.** Predominantly low-income summary statistics.

| **Variable** | **Mean** | **SD** | **Min** | **Pctile[25]** | **Pctile[75]** | **Max** |
| --- | --- | --- | --- | --- | --- | --- |
| Enrolled | 610 | 495 | 16 | 325 | 717 | 5850 |
| Absent | 53 | 65 | 0 | 23 | 62 | 3332 |
| Arrests | 8.1 | 8.3 | 0 | 2 | 12 | 173 |
| Violence | 2.1 | 2.2 | 0 | 0 | 3 | 24 |
| High School | 0.32 | 0.47 | 0 | 0 | 1 | 1 |
| Perc. Black | 33 | 27 | 0 | 9.8 | 53 | 99 |
| Perc. Poverty | 81 | 12 | 50 | 74 | 91 | 100 |

*Summary statistics of schools where more than 50% of students were considered in poverty*

**Table S5.** Predominantly Black and predominantly low-income summary statistics.

| **Variable** | **Mean** | **SD** | **Min** | **Pctile[25]** | **Pctile[75]** | **Max** |
| --- | --- | --- | --- | --- | --- | --- |
| Enrolled | 433 | 247 | 16 | 253 | 556 | 2146 |
| Absent | 44 | 41 | 0 | 20 | 54 | 1464 |
| Arrests | 7.6 | 7.3 | 0 | 2 | 11 | 170 |
| Violence | 2.1 | 2.1 | 0 | 0 | 3 | 21 |
| High School | 0.3 | 0.46 | 0 | 0 | 1 | 1 |
| Perc. Black | 73 | 12 | 50 | 62 | 83 | 99 |
| Perc. Poverty | 83 | 9.7 | 52 | 76 | 91 | 100 |

*Summary statistics of schools where more than 50% of students were non-Hispanic Black and more than 50% of students were considered in poverty*

**Table S6.** Predominantly non-Black school summary statistics.

| **Variable** | **Mean** | **SD** | **Min** | **Pctile[25]** | **Pctile[75]** | **Max** |
| --- | --- | --- | --- | --- | --- | --- |
| Enrolled | 663 | 529 | 14 | 359 | 794 | 5955 |
| Absent | 53 | 67 | 0 | 22 | 60 | 3332 |
| Arrests | 7.4 | 8.1 | 0 | 1 | 11 | 246 |
| Violence | 1.9 | 2.2 | 0 | 0 | 3 | 28 |
| High School | 0.3 | 0.46 | 0 | 0 | 1 | 1 |
| Perc. Black | 14 | 14 | 0 | 0.9 | 24 | 50 |
| Perc. Poverty | 58 | 35 | 0.038 | 28 | 87 | 100 |

*Summary statistics of schools where less than 50% of students were non-Hispanic Black*

**Table S7.** Predominantly non-low-income summary statistics.

| **Variable** | **Mean** | **SD** | **Min** | **Pctile[25]** | **Pctile[75]** | **Max** |
| --- | --- | --- | --- | --- | --- | --- |
| Enrolled | 641 | 497 | 14 | 359 | 768 | 5955 |
| Absent | 44 | 54 | 0 | 19 | 52 | 3251 |
| Arrests | 5.5 | 6.8 | 0 | 1 | 8 | 246 |
| Violence | 1.6 | 2.1 | 0 | 0 | 2 | 28 |
| High School | 0.24 | 0.43 | 0 | 0 | 0 | 1 |
| Perc. Black | 3.3 | 6.9 | 0 | 0.16 | 3.2 | 83 |
| Perc. Poverty | 13 | 17 | 0.038 | 0.76 | 29 | 50 |

*Summary statistics of schools where less than 50% of students were considered in poverty*

**Table S8.** Predominantly non-Black and predominantly non-low-income summary statistics.

| **Variable** | **Mean** | **SD** | **Min** | **Pctile[25]** | **Pctile[75]** | **Max** |
| --- | --- | --- | --- | --- | --- | --- |
| Enrolled | 641 | 497 | 14 | 358 | 766 | 5955 |
| Absent | 44 | 54 | 0 | 19 | 52 | 3251 |
| Arrests | 5.5 | 6.8 | 0 | 1 | 8 | 246 |
| Violence | 1.6 | 2.1 | 0 | 0 | 2 | 28 |
| High School | 0.24 | 0.43 | 0 | 0 | 0 | 1 |
| Perc. Black | 3.1 | 6.1 | 0 | 0.16 | 3.1 | 45 |
| Perc. Poverty | 13 | 17 | 0.038 | 0.76 | 28 | 50 |

*Summary statistics of schools where less than 50% of students were non-Hispanic Black and less than 50% of students were considered in poverty*

**Table S9.** Poisson models predicting school absenteeism in all schools (All racial groups interaction)

|  | Model 1 | Model 2 | Model 3 | Model 4 | Model 5 |
| --- | --- | --- | --- | --- | --- |
| Nearby Arrests | 0.00104 *** |  | 0.00096 *** | -0.00228 ** | 0.00012 |
|  | (0.00010) |  | (0.00010) | (0.00087) | (0.00021) |
| Nearby Violence |  | 0.00177 *** | 0.00135 *** | 0.00281 | 0.00060 |
|  |  | (0.00025) | (0.00025) | (0.00296) | (0.00051) |
| Nearby Arrests X Perc. Black |  |  |  | 0.00005 *** |  |
|  |  |  |  | (0.00001) |  |
| Nearby Violence X Perc. Black |  |  |  | -0.00003 |  |
|  |  |  |  | (0.00003) |  |
| Nearby Arrests X Perc. Hispanic |  |  |  | 0.00003 ** |  |
|  |  |  |  | (0.00001) |  |
| Nearby Violence X Perc. Hispanic |  |  |  | 0.00000 |  |
|  |  |  |  | (0.00003) |  |
| Nearby Arrests X Perc. Asian |  |  |  | 0.00000 |  |
|  |  |  |  | (0.00001) |  |
| Nearby Violence X Perc. Asian |  |  |  | -0.00003 |  |
|  |  |  |  | (0.00005) |  |
| Nearby Arrests X Perc. Other |  |  |  | 0.00003 ** |  |
|  |  |  |  | (0.00001) |  |
| Nearby Violence X Perc. Other |  |  |  | -0.00002 |  |
|  |  |  |  | (0.00003) |  |
| Nearby Arrests X Perc. Poverty |  |  |  |  | 0.00001 *** |
|  |  |  |  |  | (0.00000) |
| Nearby Violence X Perc. Poverty |  |  |  |  | 0.00001 |
|  |  |  |  |  | (0.00001) |
| N | 1587887 | 1587887 | 1587887 | 1587887 | 1587887 |
| AIC | 16589216.80170 | 16590088.98576 | 16588874.58888 | 16587374.85673 | 16588638.12213 |
| BIC | 16713849.91442 | 16714722.09849 | 16713519.97952 | 16712118.47069 | 16713308.06860 |
| Pseudo R2 | 0.77118 | 0.77117 | 0.77119 | 0.77121 | 0.77119 |
| *** p < 0.001; ** p < 0.01; * p < 0.05. | | | | | |

**Table S10.** Poisson models predicting school absenteeism in all schools (Quadratic interaction)

|  | Model 1 | Model 2 | Model 3 | Model 4 | Model 5 |
| --- | --- | --- | --- | --- | --- |
| Nearby Arrests | 0.0010412 *** |  | 0.0009582 *** | -0.0011250 *** | -0.0001983 |
|  | (0.0001017) |  | (0.0001042) | (0.0001888) | (0.0002265) |
| Nearby Violence |  | 0.0017656 *** | 0.0013508 *** | 0.0019578 *** | 0.0009655 |
|  |  | (0.0002474) | (0.0002540) | (0.0004486) | (0.0005396) |
| Nearby Arrests X Perc. Black |  |  |  | 0.0001328 *** |  |
|  |  |  |  | (0.0000125) |  |
| Nearby Violence X Perc. Black |  |  |  | -0.0000473 |  |
|  |  |  |  | (0.0000306) |  |
| Nearby Arrests X Perc. Black Sq. |  |  |  | -0.0000013 *** |  |
|  |  |  |  | (0.0000002) |  |
| Nearby Violence X Perc. Black Sq. |  |  |  | 0.0000005 |  |
|  |  |  |  | (0.0000004) |  |
| Nearby Arrests X Perc. Poverty |  |  |  |  | 0.0000429 *** |
|  |  |  |  |  | (0.0000126) |
| Nearby Violence X Perc. Poverty |  |  |  |  | -0.0000375 |
|  |  |  |  |  | (0.0000340) |
| Nearby Arrests X Perc. Poverty Sq. |  |  |  |  | -0.0000003 * |
|  |  |  |  |  | (0.0000001) |
| Nearby Violence X Perc. Poverty Sq. |  |  |  |  | 0.0000005 |
|  |  |  |  |  | (0.0000004) |
| N | 1587887 | 1587887 | 1587887 | 1587887 | 1587887 |
| AIC | 16589216.8016988 | 16590088.9857619 | 16588874.5888823 | 16586894.3321506 | 16588539.0387173 |
| BIC | 16713849.9144232 | 16714722.0984863 | 16713519.9795215 | 16711588.8344488 | 16713233.5410154 |
| Pseudo R2 | 0.7711818 | 0.7711698 | 0.7711866 | 0.7712140 | 0.7711913 |
| *** p < 0.001; ** p < 0.01; * p < 0.05. | | | | | |

**Table S11.** Poisson models predicting school absenteeism in all schools (Excluding violent crime arrests)

|  | Model 1 | Model 2 | Model 3 | Model 4 | Model 5 | Model 6 |
| --- | --- | --- | --- | --- | --- | --- |
| Nearby Arrests | 0.00096 *** |  | 0.00095 *** | -0.00012 | 0.00008 | -0.00005 |
|  | (0.00012) |  | (0.00012) | (0.00016) | (0.00023) | (0.00023) |
| Nearby Violence |  | 0.00177 *** | 0.00173 *** | 0.00155 *** | 0.00065 | 0.00169 *** |
|  |  | (0.00025) | (0.00025) | (0.00035) | (0.00050) | (0.00025) |
| Nearby Arrests X Perc. Black |  |  |  | 0.00004 *** |  | 0.00004 *** |
|  |  |  |  | (0.00000) |  | (0.00000) |
| Nearby Violence X Perc. Black |  |  |  | 0.00001 |  |  |
|  |  |  |  | (0.00001) |  |  |
| Nearby Arrests X Perc. Poverty |  |  |  |  | 0.00001 *** | 0.00000 |
|  |  |  |  |  | (0.00000) | (0.00000) |
| Nearby Violence X Perc. Poverty |  |  |  |  | 0.00002 * |  |
|  |  |  |  |  | (0.00001) |  |
| N | 1587887 | 1587887 | 1587887 | 1587887 | 1587887 | 1587887 |
| AIC | 16589664.76840 | 16590088.98576 | 16589085.58117 | 16588079.33386 | 16588870.14314 | 16588081.59707 |
| BIC | 16714297.88113 | 16714722.09849 | 16713730.97181 | 16712749.28033 | 16713540.08961 | 16712751.54354 |
| Pseudo R2 | 0.77118 | 0.77117 | 0.77118 | 0.77120 | 0.77119 | 0.77120 |
| *** p < 0.001; ** p < 0.01; * p < 0.05. | | | | | | |

**Table S12.** Poisson models predicting school absenteeism in all schools (Controlling for 311 calls)

|  | Model 1 | Model 2 | Model 3 | Model 4 | Model 5 | Model 6 |
| --- | --- | --- | --- | --- | --- | --- |
| Nearby Arrests | 0.00105 *** |  | 0.00097 *** | 0.00001 | 0.00012 | -0.00004 |
|  | (0.00010) |  | (0.00010) | (0.00014) | (0.00021) | (0.00021) |
| NYPD 311 Calls | 0.00024 *** | 0.00020 ** | 0.00022 ** | 0.00022 ** | 0.00023 ** | 0.00022 ** |
|  | (0.00007) | (0.00007) | (0.00007) | (0.00007) | (0.00007) | (0.00007) |
| Nearby Violence |  | 0.00171 *** | 0.00128 *** | 0.00143 *** | 0.00051 | 0.00126 *** |
|  |  | (0.00025) | (0.00025) | (0.00036) | (0.00051) | (0.00025) |
| Nearby Arrests X Perc. Black |  |  |  | 0.00003 *** |  | 0.00003 *** |
|  |  |  |  | (0.00000) |  | (0.00000) |
| Nearby Violence X Perc. Black |  |  |  | -0.00001 |  |  |
|  |  |  |  | (0.00001) |  |  |
| Nearby Arrests X Perc. Poverty |  |  |  |  | 0.00001 *** | 0.00000 |
|  |  |  |  |  | (0.00000) | (0.00000) |
| Nearby Violence X Perc. Poverty |  |  |  |  | 0.00001 |  |
|  |  |  |  |  | (0.00001) |  |
| N | 1587887 | 1587887 | 1587887 | 1587887 | 1587887 | 1587887 |
| AIC | 16589004.23215 | 16589948.01311 | 16588699.28630 | 16587710.32295 | 16588456.12133 | 16587713.66311 |
| BIC | 16713649.62279 | 16714593.40375 | 16713356.95485 | 16712392.54733 | 16713138.34571 | 16712395.88749 |
| Pseudo R2 | 0.77118 | 0.77117 | 0.77119 | 0.77120 | 0.77119 | 0.77120 |
| *** p < 0.001; ** p < 0.01; * p < 0.05. | | | | | | |

**Table S13.** Poisson models predicting school absenteeism in all schools (OLS specification)

|  | Model 1 | Model 2 | Model 3 | Model 4 | Model 5 | Model 6 |
| --- | --- | --- | --- | --- | --- | --- |
| Nearby Arrests | 0.00002 * |  | 0.00001 | 0.00004 *** | 0.00007 *** | 0.00006 *** |
|  | (0.00001) |  | (0.00001) | (0.00001) | (0.00002) | (0.00002) |
| Nearby Violence |  | 0.00017 *** | 0.00016 *** | 0.00011 *** | 0.00009 | 0.00016 *** |
|  |  | (0.00002) | (0.00002) | (0.00003) | (0.00004) | (0.00002) |
| Nearby Arrests X Perc. Black |  |  |  | -0.00000 *** |  | -0.00000 * |
|  |  |  |  | (0.00000) |  | (0.00000) |
| Nearby Violence X Perc. Black |  |  |  | 0.00000 * |  |  |
|  |  |  |  | (0.00000) |  |  |
| Nearby Arrests X Perc. Poverty |  |  |  |  | -0.00000 *** | 0.00000 |
|  |  |  |  |  | (0.00000) | (0.00000) |
| Nearby Violence X Perc. Poverty |  |  |  |  | 0.00000 |  |
|  |  |  |  |  | (0.00000) |  |
| N | 1587893 | 1587893 | 1587893 | 1587893 | 1587893 | 1587893 |
| AIC | -5852959.88748 | -5853027.70247 | -5853026.96909 | -5853044.45865 | -5853039.62644 | -5853041.80269 |
| BIC | -5728314.45849 | -5728382.27347 | -5728369.26217 | -5728362.19589 | -5728357.36369 | -5728359.53993 |
| Pseudo R2 |  |  |  |  |  |  |
| *** p < 0.001; ** p < 0.01; * p < 0.05. | | | | | | |

**Table S14.** Poisson models predicting school absenteeism in all schools (Standardizing arrests and violence by school)

|  | Model 1 | Model 2 | Model 3 | Model 4 | Model 5 | Model 6 |
| --- | --- | --- | --- | --- | --- | --- |
| Nearby Arrests | 0.00247 *** |  | 0.00206 *** | -0.00099 | -0.00124 | -0.00141 |
|  | (0.00048) |  | (0.00050) | (0.00070) | (0.00105) | (0.00102) |
| Nearby Violence |  | 0.00224 *** | 0.00186 *** | 0.00174 ** | 0.00090 | 0.00185 *** |
|  |  | (0.00044) | (0.00045) | (0.00062) | (0.00092) | (0.00045) |
| Nearby Arrests X Perc. Black |  |  |  | 0.00013 *** |  | 0.00013 *** |
|  |  |  |  | (0.00002) |  | (0.00002) |
| Nearby Violence X Perc. Black |  |  |  | 0.00000 |  |  |
|  |  |  |  | (0.00002) |  |  |
| Nearby Arrests X Perc. Poverty |  |  |  |  | 0.00005 *** | 0.00001 |
|  |  |  |  |  | (0.00001) | (0.00002) |
| Nearby Violence X Perc. Poverty |  |  |  |  | 0.00002 |  |
|  |  |  |  |  | (0.00001) |  |
| N | 1586892 | 1586538 | 1586538 | 1586538 | 1586538 | 1586538 |
| AIC | 16580797.45589 | 16577830.09570 | 16577560.70515 | 16576718.84900 | 16577310.91231 | 16576715.84779 |
| BIC | 16705350.54208 | 16702356.36439 | 16702099.25091 | 16701281.94888 | 16701874.01219 | 16701278.94768 |
| Pseudo R2 | 0.77125 | 0.77126 | 0.77126 | 0.77127 | 0.77126 | 0.77127 |
| *** p < 0.001; ** p < 0.01; * p < 0.05. | | | | | | |

**Table S15.** Poisson models predicting school absenteeism in all schools (500-meter bandwidth)

|  | Model 1 | Model 2 | Model 3 | Model 4 | Model 5 | Model 6 |
| --- | --- | --- | --- | --- | --- | --- |
| Nearby Arrests | 0.00138 *** |  | 0.00126 *** | 0.00015 | 0.00018 | 0.00002 |
|  | (0.00019) |  | (0.00019) | (0.00027) | (0.00037) | (0.00037) |
| Nearby Violence |  | 0.00246 *** | 0.00191 *** | 0.00192 ** | 0.00079 | 0.00191 *** |
|  |  | (0.00047) | (0.00048) | (0.00069) | (0.00095) | (0.00048) |
| Nearby Arrests X Perc. Black |  |  |  | 0.00004 *** |  | 0.00004 *** |
|  |  |  |  | (0.00001) |  | (0.00001) |
| Nearby Violence X Perc. Black |  |  |  | 0.00000 |  |  |
|  |  |  |  | (0.00002) |  |  |
| Nearby Arrests X Perc. Poverty |  |  |  |  | 0.00002 ** | 0.00000 |
|  |  |  |  |  | (0.00000) | (0.00001) |
| Nearby Violence X Perc. Poverty |  |  |  |  | 0.00002 |  |
|  |  |  |  |  | (0.00001) |  |
| N | 1587887 | 1587887 | 1587887 | 1587887 | 1587887 | 1587887 |
| AIC | 16590031.35643 | 16590372.08334 | 16589844.85655 | 16589502.77113 | 16589726.91443 | 16589500.96717 |
| BIC | 16714664.46915 | 16715005.19607 | 16714490.24719 | 16714172.71760 | 16714396.86090 | 16714170.91364 |
| Pseudo R2 | 0.77117 | 0.77117 | 0.77117 | 0.77118 | 0.77117 | 0.77118 |
| *** p < 0.001; ** p < 0.01; * p < 0.05. | | | | | | |

**Table S16.** Poisson models predicting school absenteeism in all schools (250-meter bandwidth)

|  | Model 1 | Model 2 | Model 3 | Model 4 | Model 5 | Model 6 |
| --- | --- | --- | --- | --- | --- | --- |
| Nearby Arrests | 0.00110 ** |  | 0.00084 * | -0.00059 | -0.00064 | -0.00091 |
|  | (0.00037) |  | (0.00037) | (0.00049) | (0.00058) | (0.00059) |
| Nearby Violence |  | 0.00400 *** | 0.00366 *** | 0.00347 ** | 0.00115 | 0.00362 *** |
|  |  | (0.00085) | (0.00087) | (0.00130) | (0.00169) | (0.00087) |
| Nearby Arrests X Perc. Black |  |  |  | 0.00005 *** |  | 0.00005 ** |
|  |  |  |  | (0.00001) |  | (0.00002) |
| Nearby Violence X Perc. Black |  |  |  | 0.00001 |  |  |
|  |  |  |  | (0.00003) |  |  |
| Nearby Arrests X Perc. Poverty |  |  |  |  | 0.00002 ** | 0.00001 |
|  |  |  |  |  | (0.00001) | (0.00001) |
| Nearby Violence X Perc. Poverty |  |  |  |  | 0.00004 |  |
|  |  |  |  |  | (0.00002) |  |
| N | 1587887 | 1587887 | 1587887 | 1587887 | 1587887 | 1587887 |
| AIC | 16590598.16797 | 16590470.34990 | 16590419.04988 | 16590272.23923 | 16590344.98817 | 16590269.74083 |
| BIC | 16715231.28070 | 16715103.46262 | 16715064.44052 | 16714942.18570 | 16715014.93463 | 16714939.68730 |
| Pseudo R2 | 0.77116 | 0.77116 | 0.77117 | 0.77117 | 0.77117 | 0.77117 |
| *** p < 0.001; ** p < 0.01; * p < 0.05. | | | | | | |
